# Deep Learning–Based Localization and Detection of Malpositioned Nasogastric Tubes on Portable Supine Chest X-Rays in Intensive Care and Emergency Medicine: A Multi-center Retrospective Study

**DOI:** 10.1007/s10278-024-01181-z

**Published:** 2024-07-09

**Authors:** Chih-Hung Wang, Tianyu Hwang, Yu-Sen Huang, Joyce Tay, Cheng-Yi Wu, Meng-Che Wu, Holger R. Roth, Dong Yang, Can Zhao, Weichung Wang, Chien-Hua Huang

**Affiliations:** 1 Department of Emergency Medicine, College of Medicine, National Taiwan University, Taipei, Taiwan; 2 Department of Emergency Medicine, Zhongzheng Dist, National Taiwan University Hospital, No. 7, Zhongshan S. Rd, Taipei City 100, Taiwan; 3 Mathematics Division, National Center for Theoretical Sciences, National Taiwan University, Taipei, Taiwan; 4 Department of Medical Imaging, National Taiwan University Hospital, Taipei, Taiwan; 5 NVIDIA Corporation, Bethesda, CA USA; 6 Institute of Applied Mathematical Sciences, National Taiwan University, No. 1, Sec. 4, Roosevelt Rd, Taipei, 106 Taiwan

**Keywords:** Artificial intelligence, Chest radiograph, Chest X-ray, Deep learning, Malposition, Misplacement, Nasogastric tube

## Abstract

Malposition of a nasogastric tube (NGT) can lead to severe complications. We aimed to develop a computer-aided detection (CAD) system to localize NGTs and detect NGT malposition on portable chest X-rays (CXRs). A total of 7378 portable CXRs were retrospectively retrieved from two hospitals between 2015 and 2020. All CXRs were annotated with pixel-level labels for NGT localization and image-level labels for NGT presence and malposition. In the CAD system, DeepLabv3 + with backbone ResNeSt50 and DenseNet121 served as the model architecture for segmentation and classification models, respectively. The CAD system was tested on images from chronologically different datasets (National Taiwan University Hospital (National Taiwan University Hospital)-20), geographically different datasets (National Taiwan University Hospital-Yunlin Branch (YB)), and the public CLiP dataset. For the segmentation model, the Dice coefficients indicated accurate delineation of the NGT course (National Taiwan University Hospital-20: 0.665, 95% confidence interval (CI) 0.630–0.696; National Taiwan University Hospital-Yunlin Branch: 0.646, 95% CI 0.614–0.678). The distance between the predicted and ground-truth NGT tips suggested accurate tip localization (National Taiwan University Hospital-20: 1.64 cm, 95% CI 0.99–2.41; National Taiwan University Hospital-Yunlin Branch: 2.83 cm, 95% CI 1.94–3.76). For the classification model, NGT presence was detected with high accuracy (area under the receiver operating characteristic curve (AUC): National Taiwan University Hospital-20: 0.998, 95% CI 0.995–1.000; National Taiwan University Hospital-Yunlin Branch: 0.998, 95% CI 0.995–1.000; CLiP dataset: 0.991, 95% CI 0.990–0.992). The CAD system also detected NGT malposition with high accuracy (AUC: National Taiwan University Hospital-20: 0.964, 95% CI 0.917–1.000; National Taiwan University Hospital-Yunlin Branch: 0.991, 95% CI 0.970–1.000) and detected abnormal nasoenteric tube positions with favorable performance (AUC: 0.839, 95% CI 0.807–0.869). The CAD system accurately localized NGTs and detected NGT malposition, demonstrating excellent potential for external generalizability.

**Supplementary Information:**

The online version contains supplementary material available at 10.1007/s10278-024-01181-z.

## Introduction

A nasogastric tube (NGT) is a flexible rubber or plastic tube that is passed through the nostril, down the esophagus, and into the stomach. It may be indicated for various scenarios such as gastric decompression or administration of medications in intensive care units (ICUs) or emergency departments (EDs).

A malpositioned NGT in the main bronchus of the lung may lead to complications such as pneumonia, respiratory failure, and death [1]. The prevalence of NGT placement errors in adults has been estimated to vary from 1.3% [2] to 89.5% [3], depending on the error definition. In the UK, feeding through a misplaced NGT is recognized by the National Patient Safety Agency (NPSA) of the National Health Service [4] as a serious patient safety issue that is “wholly preventable if guidance or safety recommendations that provide strong systemic protective barriers are available at a national level [5].”

A chest X-ray (CXR) is considered the gold standard in verifying NGT position [6], and the importance of the radiologist’s role in verification has been emphasized by the NPSA [7, 8]. Between April 2021 and March 2022, a total of 31 incidents in which feeding through a misplaced NGT occurred were reported to the NPSA [9]; among these, CXR misinterpretation accounted for 14 (45%) and was ranked as the most frequently encountered mistake [9]. For patients in ICUs or EDs, CXRs are predominantly obtained using a portable X-ray machine. However, Torsy et al. [10] showed that in 16.9% of portable CXRs, the image quality was insufficient to conclusively determine the NGT position. While well-trained radiologists are crucial for confirming NGT placement, they may not always be readily available.

Few studies have employed deep learning to localize an NGT and detect its malposition. Most previous studies [11–13] focused on detecting NGT presence, using small datasets of 25–107 images. These models employed outdated image processing techniques [14, 15], which may fail when the NGT forms a loop or similar objects are present [13]. Consequently, they [11–13] achieved only moderate performance. Singh et al. [16] used 5475 radiographs to develop deep learning models (Inception V3, ResNet50, DenseNet121) for detecting bronchial insertion of an NGT. When tested on 100 images, Inception V3 showed the highest AUC (0.87) with a sensitivity of 0.88 and specificity of 0.76.

To the best of our knowledge, there is a lack of deep learning models capable of localizing an NGT and simultaneously detecting its malposition. Therefore, this study aimed to develop a deep learning–based computer-aided detection (CAD) system to assist in the localization of NGTs and identify their malposition on portable supine CXRs.

## Materials and Methods

### Study Design and Setting

This retrospective study received approval from the Research Ethics Committee of National Taiwan University Hospital (reference number: 202003106RINC), with a waiver of consent granted. Portable supine CXRs were obtained from the Picture Archiving and Communication System (PACS) database of National Taiwan University Hospital and its Yunlin Branch. The study findings are presented in accordance with the Checklist for Artificial Intelligence in Medical Imaging (CLAIM) [17]. The methods in building the datasets, annotating the images, and developing the CAD system have been detailed in previous studies [18–20] for different study purposes.

### Image Acquisition and Dataset Construction

**Fig. 1:**
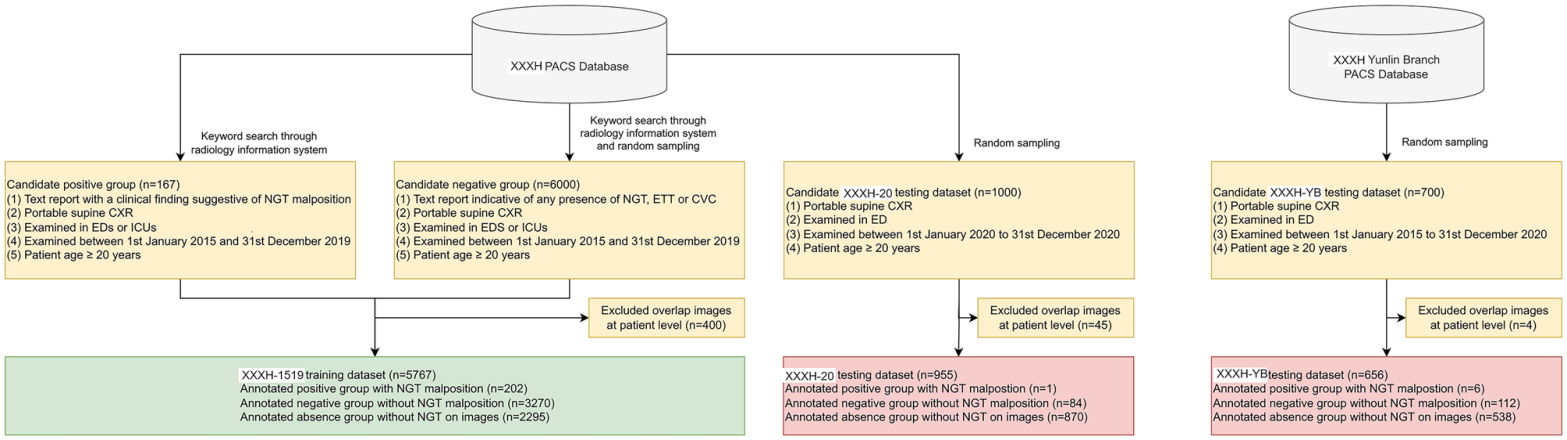
Flowchart of the image inclusion process and dataset designation. CVC, central venous catheter; CXR, chest X-ray; ED, emergency department; ETT, endotracheal tube; ICU, intensive care unit; NGT, nasogastric tube; NTUH, National Taiwan University Hospital; NTUH-YB, National Taiwan University Hospital-Yunlin Branch; PACS, Picture Archiving and Communication System

A radiology information system was employed to search the PACS databases for candidate CXRs (Fig. 1). Candidate positive images that showed NGT malposition were identified through a keywords search (Supplemental Table 1). The inclusion criteria for these images were as follows: (1) had text report of NGT malposition, (2) was a portable supine CXR, (3) obtained in EDs or ICUs, (4) examined between 1 January 2015 and 31 December 2019, and (5) patient age ≥ 20 years. The inclusion criteria of the candidate group negative for NGT malposition were the same as the candidate positive group, except that the text reports of the candidate negative group had to indicate the presence of at least one of the following devices: NGT, central venous catheter (CVC), or endotracheal tube (ETT). Because the number of images without NGT malposition was far greater than those with NGT malposition, we randomly selected 6000 images that met the inclusion criteria of the candidate negative group. The selected list of the candidate groups was further examined to avoid overlap of the positive and negative groups; i.e., for each patient, only one image was selected for analysis. After duplicate images were excluded, the positive and negative groups comprised the National Taiwan University Hospital-1519 training dataset for model development.

**Table 1:** Basic characteristics of the training and testing datasets

Variables	National Taiwan University Hospital-1519 training dataset (*n* = 5767)	National Taiwan University Hospital-20 testing dataset (*n* = 955)	National Taiwan University Hospital-Yunlin Branch testing dataset (*n* = 656)	*p* value
Age, years	65.6 (16.7)	57.9 (20.2)	67.6 (20.0)	< 0.001
Male, *n*	3460 (60.0)	496 (51.9)	381 (58.1)	< 0.001
Annotated positive images, *n*	202 (3.5)	1 (0.1)	6 (0.9)	< 0.001
Bronchial insertion	12 (0.2)	0 (0)	1 (0.2)	0.361
Esophageal insertion	190 (3.3)	1 (0.1)	5 (0.8)	< 0.001
Annotated negative images, *n*	3270 (56.7)	84 (8.8)	112 (17.1)	< 0.001
Annotated absence images, *n*	2295 (39.8)	870 (91.1)	538 (82.0)	< 0.001

Data are presented as mean (standard deviation) or counts (proportion)

In order to test the performance of the CAD system in a simulated real-world setting, a random sampling method was used to construct the testing datasets, National Taiwan University Hospital-20 and National Taiwan University Hospital-Yunlin Branch. As suggested by the guidelines [21], external validation can involve data collected by the same team, using identical predictors and outcome definitions, but typically sampled from a different timeframe (temporal validation) or setting (geographical validation). In our study, the National Taiwan University Hospital-20 dataset included CXRs taken in 2020 at National Taiwan University Hospital, while the National Taiwan University Hospital-Yunlin Branch dataset comprised CXRs from 2015 to 2020 at National Taiwan University Hospital-Yunlin Branch. Compared to NTUH-1519, NTUH-20 was a dataset from a different period (2015–2019 vs. 2020), and NTUH-YB was from a different location (NTUH vs. NTUH-YB). According to the guidelines [21], these temporally and geographically distinct datasets can be used to assess the external generalizability of the CAD system. All eligible images were exported in Digital Imaging and Communications in Medicine format for annotation.

The Catheter and Line Position (CLiP) dataset developed by Tang et al. [22] provided CXRs selected from the NIH ChestXray14 dataset [23]. The CLiP dataset includes CXRs with NGTs as well as nasoenteric tubes, such as nasojejunal and nasoduodenal tubes. These CXRs were obtained from individuals older than 10 years, and not all images were taken with portable CXR machines. Tang et al. [22] defined any nasoenteric tube within the airway system, the esophagus, or coiled anywhere above the gastroesophageal sphincter as being in an abnormal position. The dataset includes 30,083 CXRs from 3791 patients with a median age of 49; among these, 267 (0.9%) have abnormally positioned nasoenteric tubes. Few public datasets are available to examine NGT position on portable anteroposterior CXRs. Although the CXRs in the CLiP dataset did not meet the inclusion criteria for our study, they may still serve as a resource for external validation to some extent.

### Image Annotation and Ground Truth

For segmentation tasks, a sequential annotation procedure was employed. Each image was first randomly assigned to nurse practitioners, who added pixel-level labels for the NGT, NGT tip, lung, and diaphragm. These annotated images were then randomly assigned to emergency medicine (EM) physicians for review and adjustment if necessary.

For classification tasks, each image was classified based on the presence and position of the NGT. According to clinicians’ discretion, images were annotated with image-level labels as either positive for malpositioned NGT, negative for correctly positioned NGT, or absence group with no NGT visible. NGT malposition was further categorized as bronchial or esophageal. Each image was randomly assigned to one EM senior resident and one EM attending physician for annotation, with both annotators blind to each other’s results.

A total of ten nurse practitioners, eight EM senior residents, and eight EM attending physicians participated in the annotation process, each with a minimum of 4 years of clinical experience. All annotated images for both segmentation and classification tasks were reviewed by a thoracic radiologist with 15 years of clinical experience for final approval and used as the ground truth.

### Development of the Algorithm

**Fig. 2:**
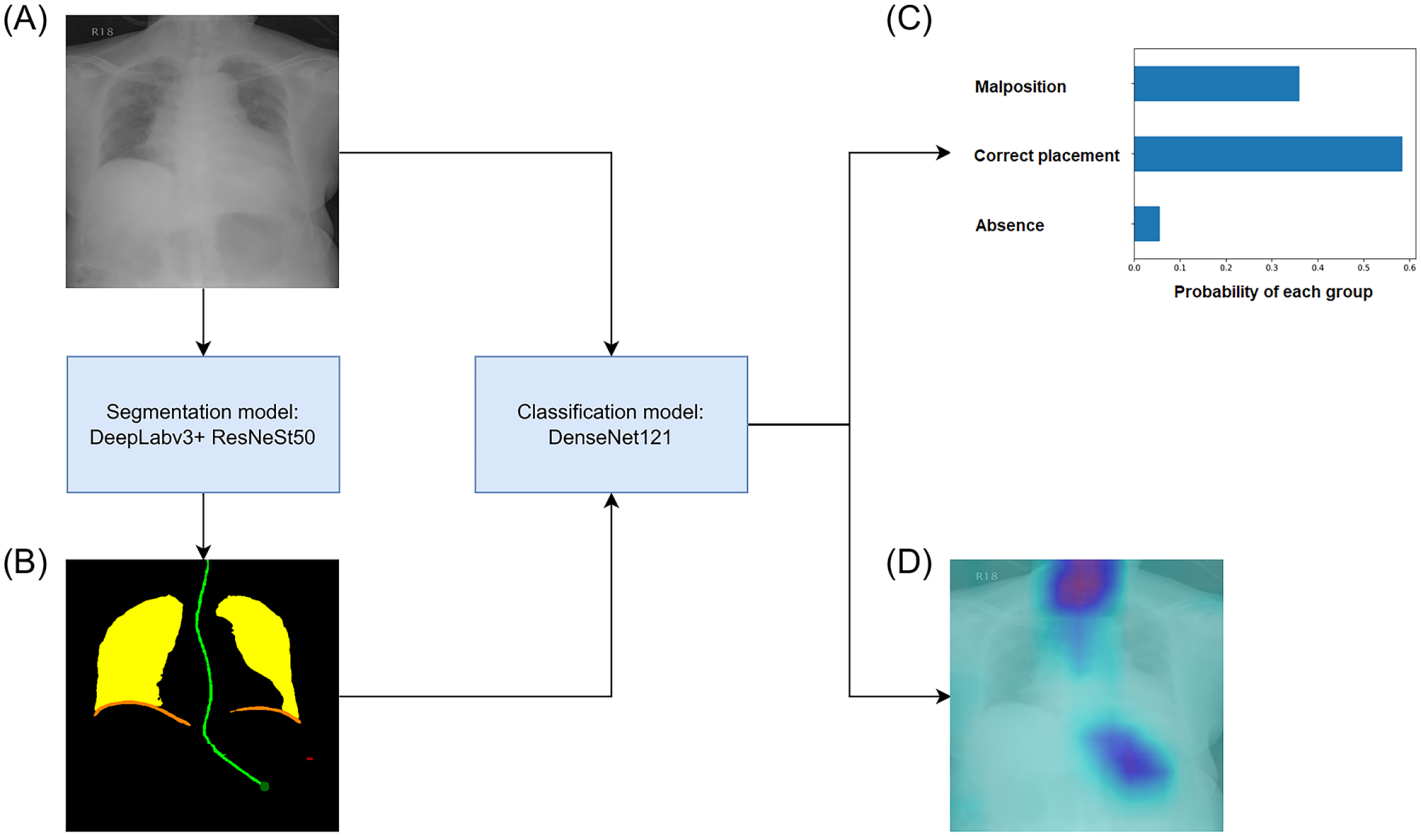
The two-stage pipeline of the CAD system. The CAD system comprised a segmentation model and a classification model. DeepLabv3 + with ResNeSt50 backbone served as the basic architecture of the segmentation model, and DenseNet121 was the basic architecture for the classification model. The input of CXR image (**A**) was first passed into the segmentation model to derive the segmentation masks of NGT, NGT tip, lung, and diaphragm (**B**). The segmentation masks (**B**) were then passed into the classification model along with the input image (**A**) to assess NGT positioning, providing the probabilities of NGT presence, NGT malposition, and NGT absence (**C**). Concurrently, the classification model produced CAM (**D**) to visualize and explain the classification result. CAD, computer-aided detection; CXR, chest X-ray; NGT, nasogastric tube; CAM, class activation mapping

Following annotation, the National Taiwan University Hospital-1519 dataset was randomly divided into five subgroups (folds) ensuring similar numbers of annotated images across all strata to develop the model. As shown in Fig. 2, the CAD system consisted of two models, where the first model was trained to segment out NGT, NGT tip, lung, and diaphragm (segmentation model); these segmentation masks then served as input to help detect the presence as well as malposition of the NGT (classification model).

**Fig. 3:**
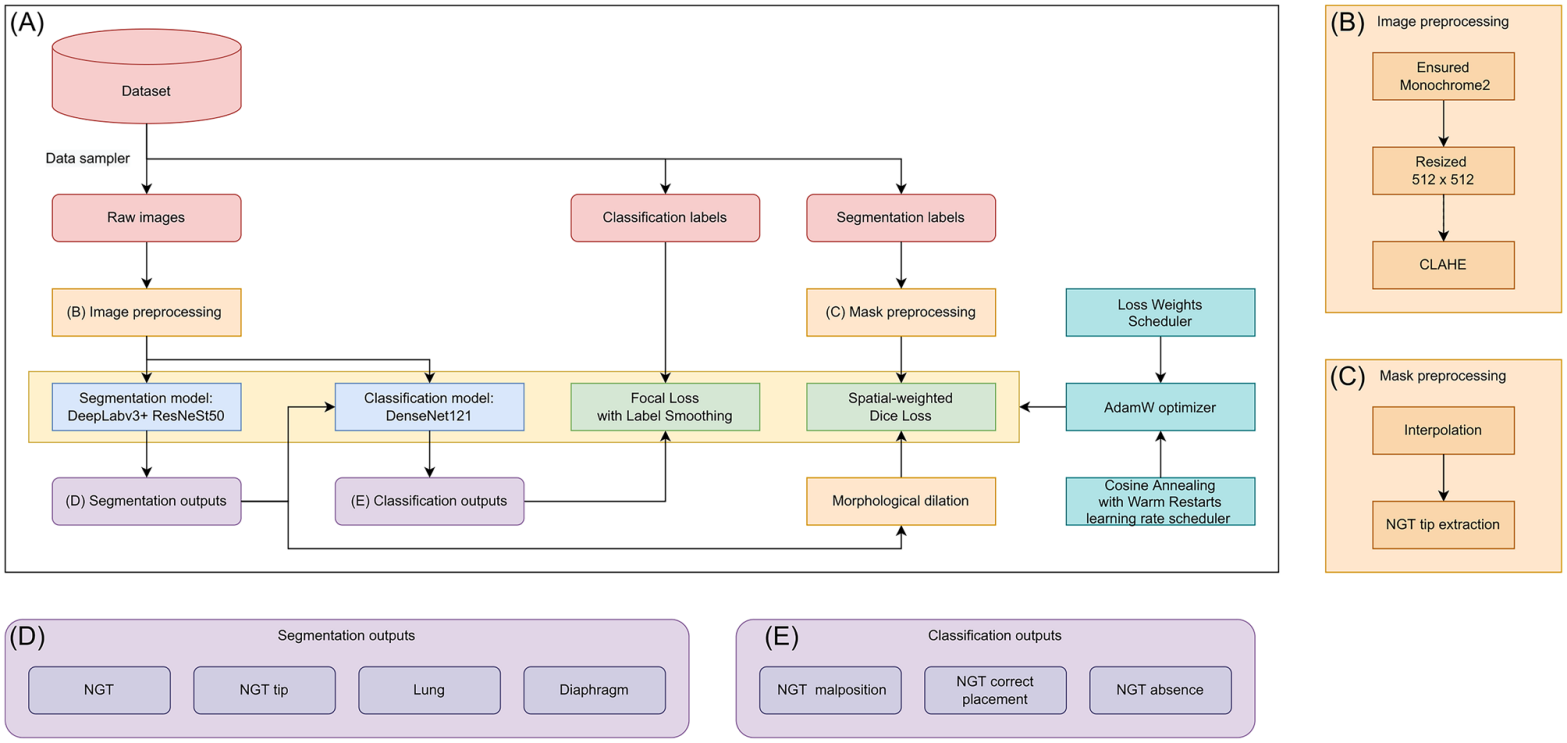
The training pipeline for the CAD system (**A**). CXR images and ground-truth labels (red rounded rectangle) were sampled from the dataset (red rounded rectangle). Images and labels then underwent preprocessing (orange rectangle) for training (yellow rectangle). In preprocessing (**B**), images were ensured to be Monochrome2, resized to 512 × 512 pixels, and transformed by CLAHE. In preprocessing (**C**), segmentation labels were interpolated into masks; then, NGT tips were extracted. After preprocessing, images were passed into the segmentation model with DeepLabv3 + and ResNeSt50 backbone as the model architecture (blue rectangle). The segmentation model produced segmentation masks of NGT, NGT tip, lung, and diaphragm (**D**). These segmentation masks and preprocessed images were passed into the classification model (blue rectangle) to detect NGT presence and NGT malposition (**E**). Loss functions were used to supervise the learning process, including spatial-weighted Dice loss for segmentation and focal loss with label smoothing for classification (green rectangle). Segmentation masks were preprocessed by morphological dilation (orange rectangle) before being passed into loss functions. The training algorithm included AdamW as an optimizer, Cosine Annealing with Warm Restarts as a learning rate scheduler, and Loss Weights Scheduler as a mechanism to dynamically weight losses (cyan rectangle). In the flowchart, the rectangle is used to specify a procedure or algorithm, while the rounded rectangle is used to specify the data. CAD, computer-aided detection; CXR, chest X-ray; CLAHE, contrast limited adaptive histogram equalization; NGT, nasogastric tube

Figure 3 shows the training pipeline. In preprocessing, images were first ensured to have a photometric interpretation of Monochrome2, then resized to 512 × 512 pixels, and finally transformed by contrast limited adaptive histogram equalization (CLAHE) [24]. DeepLabv3 + [25] with backbone ResNeSt50 [26] and DenseNet121 [27] were selected as the model architecture for segmentation and classification models, respectively. The training process used a batch size of 36, the AdamW [28] optimizer, and a learning rate of 3e^−4^, adjusted by Cosine Annealing with Warm Restarts [29]. Images with NGT malposition were oversampled to balance the image number across each annotated group.

The NGT tip was assumed to play a critical role in detecting NGT malposition. Therefore, we modified Dice loss [30] to be spatially weighted, assigning more weight to pixels around the NGT tip during model training. Two loss functions supervised the learning process: spatial-weighted Dice loss [30] for segmentation and focal loss [31] with label smoothing [32] for classification. The losses were dynamically weighted to optimize the segmentation model before the classification model. The training procedure was halted after 32 epochs.

The best parameters for the National Taiwan University Hospital-1519 dataset were obtained through fivefold validation and later used for model ensembling in testing datasets. Gradient-weighted class activation mapping (CAM) [33] was employed to inspect the image areas activated by the network and understand how the algorithm made inferences.

The model was trained on an Ubuntu 16.04.7 LTS operating system, using the PyTorch 1.12.1 deep learning framework [34] with CUDA 11.6. The training utilized four Intel(R) Xeon(R) CPU E5-2650 v4 @ 2.20 GHz processors, 256 GB of hard disk space, 16 GB of RAM, and an Nvidia Titan V graphics processing unit (Nvidia Corporation, Santa Clara, CA, USA).

### Evaluation Metrics of the Algorithm

The segmentation model’s performance was measured using the Dice coefficient, which is calculated as twice the overlap area divided by the sum of the pixels in both the predicted and ground-truth masks. Additionally, the accuracy of NGT tip localization was evaluated by measuring the absolute distance between the predicted and ground-truth NGT tips (tip-tip distance).

The classification model’s performance was evaluated using the area under the receiver operating characteristics curve (AUC) and the area under the precision-recall curve. Other metrics included sensitivity, specificity, positive predictive value, and negative predictive value. The optimal threshold for these evaluation metrics was determined using Youden’s index [35] from the National Taiwan University Hospital-1519 dataset.

### Statistical Analysis

Continuous variables are shown as mean and standard deviation, while categorical variables are displayed as counts and percentages. Comparisons of continuous variables were conducted using the analysis of variance (ANOVA) test, and categorical variables were compared using the chi-squared test. The kappa coefficient was calculated to evaluate inter-annotator agreement for classifying NGT malposition. All statistical measures are reported with point estimates and 95% confidence intervals (CIs), derived using the bootstrap method with 1000 repetitio ns. All statistical analyses were carried out with SciPy version 1.8.1 [36].

## Results

As depicted in Fig. 1, a total of 7378 images were retrieved from the PACS database, with 5767 images designated for training and 1611 images for testing. Table 1 highlights significant differences between the training and testing datasets.

**Fig. 4:**
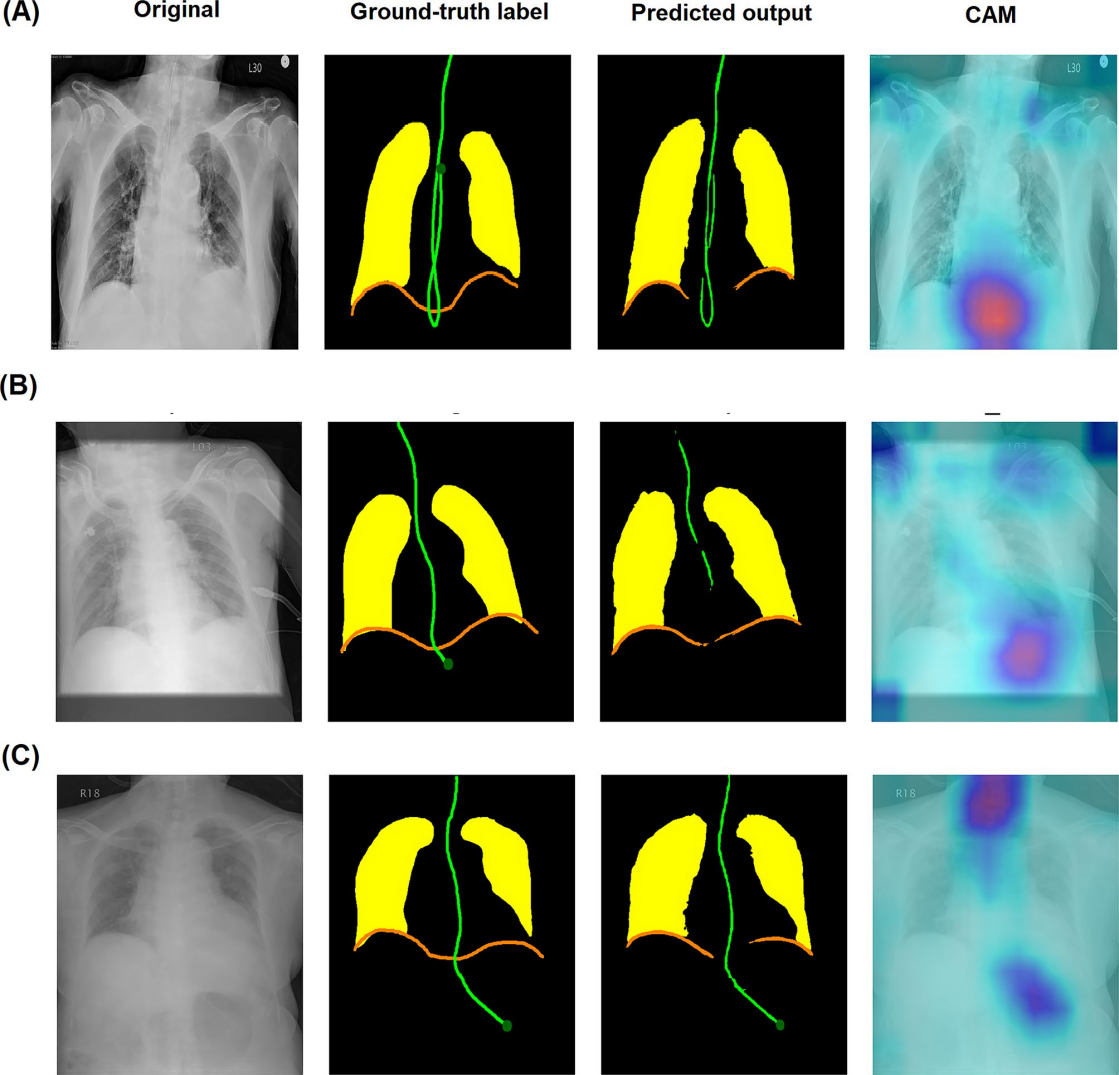
Example images stratified by prediction results of the classification model, including **A** true-positive, **B** false-positive, and **C** true-negative results. There were no false-negative cases in the testing results. The first left column presents the original images. The second left column shows the pixel-level label used by annotators and includes nasogastric tube (green), lung (yellow), and diaphragm (orange). The dark green dot represents the nasogastric tube tip. The third left column demonstrates the segmentation mask output by the segmentation model. The fourth left column shows the results of gradient-weighted class activation mapping (CAM), which reveals that the areas around the nasogastric tube tip were used by the classification model to make inferences

Figure 4 showcases three sets of representative images, with overlaid segmentation masks designed to aid in verifying NGT positions. According to Table 2, the Dice coefficient indicated that the segmentation model could accurately outline the NGT (National Taiwan University Hospital-20: 0.665, 95% CI 0.630–0.696; National Taiwan University Hospital-Yunlin Branch: 0.646, 95% CI 0.614–0.678) and lung, although its performance in identifying the diaphragm was less optimal. The tip-tip distance further demonstrated accurate localization of the NGT tip (National Taiwan University Hospital-20: 1.64 cm, 95% CI 0.99–2.41; National Taiwan University Hospital-Yunlin Branch: 2.83 cm, 95% CI 1.94–3.76).

**Table 2:** Performance of the segmentation model on the testing datasets

Dataset	Dice coefficient: NGT^a^	Dice coefficient: lung	Dice coefficient: diaphragm	Tip-tip distance, cm^a^
National Taiwan University Hospital-20	0.665 (0.630, 0.696)	0.929 (0.926, 0.931)	0.427 (0.416, 0.437)	1.64 (0.99, 2.41)
National Taiwan University Hospital-Yunlin Branch	0.646 (0.614, 0.678)	0.921 (0.918, 0.924)	0.433 (0.420, 0.446)	2.83 (1.94, 3.76)

Data are presented as point estimates (95% confidence interval)

^a^These metrics were tested on images where both predicted and ground-truth nasogastric tubes were available

Table 3 shows that the model could classify the presence of the NGT with high accuracy (AUC: National Taiwan University Hospital-20: 0.998, 95% CI 0.995–1.000; National Taiwan University Hospital-Yunlin Branch: 0.998, 95% CI 0.995–1.000; CLiP dataset: 0.991, 95% CI 0.990–0.992). Additionally, for images containing an NGT, the classification model demonstrated high accuracy in detecting NGT malposition (AUC: National Taiwan University Hospital-20: 0.964, 95% CI 0.917–1.000; National Taiwan University Hospital-Yunlin Branch: 0.991, 95% CI 0.970–1.000), effectively identifying both bronchial and esophageal insertions. In the CLiP dataset, the model also performed well in detecting abnormal positions of nasoenteric tubes (AUC 0.839, 95% CI 0.807–0.869). The CAM analysis indicated that the areas around the NGT tip were the primary focus of the classification model (Fig. 4). Table 4 also shows substantial agreement between annotators regarding NGT malposition [37].

**Table 3:** Performance of the classification model on the testing datasets

Dataset	AUC	AUPRC	Sensitivity	Specificity	PPV	NPV
Detecting NGT presence						
National Taiwan University Hospital-20	0.998 (0.995, 1.000)	0.964 (0.914, 0.998)	0.988 (0.960, 1.000)	0.989 (0.981, 0.995)	0.894 (0.828, 0.955)	0.999 (0.996, 1.000)
National Taiwan University Hospital-Yunlin Branch	0.998 (0.995, 1.000)	0.984 (0.945, 1.000)	0.992 (0.974, 1.000)	0.991 (0.981, 0.998)	0.959 (0.919, 0.992)	0.998 (0.994, 1.000)
CLiP dataset	0.991 (0.990, 0.992)	0.982 (0.980, 0.984)	0.905 (0.899, 0.911)	0.981 (0.979, 0.983)	0.947 (0.943, 0.952)	0.964 (0.962, 0.967)
Detecting NGT malposition^a^						
National Taiwan University Hospital-20	0.964 (0.917, 1.000)	0.250 (0.143, 1.000)	1.000 (1.000, 1.000)	0.964 (0.917, 1.000)	0.250 (0.143, 1.000)	1.000 (1.000, 1.000)
National Taiwan University Hospital-Yunlin Branch	0.991 (0.970, 1.000)	0.872 (0.583, 1.000)	1.000 (1.000, 1.000)	0.893 (0.833, 0.947)	0.333 (0.118, 0.579)	1.000 (1.000, 1.000)
Detecting bronchial insertion^a^						
National Taiwan University Hospital-20	NA	NA	NA	NA	NA	NA
National Taiwan University Hospital-Yunlin Branch	0.983 (0.957, 1.000)	0.333 (0.167, 1.000)	1.000 (1.000, 1.000)	0.855 (0.786, 0.915)	0.056 (0.040, 0.200)	1.000 (1.000, 1.000)
Detecting esophageal insertion^a^						
National Taiwan University Hospital-20	0.964 (0.917, 1.000)	0.250 (0.143, 1.000)	1.000 (1.000, 1.000)	0.964 (0.917, 1.000)	0.250 (0.143, 1.000)	1.000 (1.000, 1.000)
National Taiwan University Hospital-Yunlin Branch	0.984 (0.954, 1.000)	0.753 (0.333, 1.000)	1.000 (1.000, 1.000)	0.885 (0.823, 0.939)	0.278 (0.095, 0.522)	1.000 (1.000, 1.000)
Detecting abnormal position of nasoenteric tube						
CLiP dataset	0.839 (0.807, 0.869)	0.311 (0.252, 0.368)	0.738 (0.691, 0.790)	0.869 (0.861, 0.876)	0.163 (0.142, 0.183)	0.990 (0.987, 0.992)

*AUC* area under the receiver operating characteristics curve, *AUPRC* area under the precision-recall curve, *NA* not available, *NPV* negative predictive value, *PPV* positive predictive value

Data are presented as point estimates (95% confidence interval). There were no bronchial insertions in National Taiwan University Hospital-20; therefore, the relevant evaluation metrics were not able to be calculated

^a^These metrics were tested on images where ground-truth nasogastric tubes were available

**Table 4:** Interrater agreement between two annotators

Dataset	Kappa coefficient: NGT malposition	Kappa coefficient: bronchial insertion	Kappa coefficient: esophageal insertion
National Taiwan University Hospital-1519 training dataset	0.821 (0.775, 0.864)	0.267 (0.120, 0.406)	0.737 (0.674, 0.790)
National Taiwan University Hospital-20 testing dataset	1.000 (1.000, 1.000)	N/A	1.000 (1.000, 1.000)
National Taiwan University Hospital-YB testing dataset	1.000 (1.000, 1.000)	1.000 (1.000, 1.000)	1.000 (1.000, 1.000)

Data are presented as point estimates (95% confidence interval)

## Discussion

### Image Annotation

Compared to previous research [11–13, 16], our study incorporated the largest dataset of images annotated with pixel-level labels. This extensive annotation contributed to achieving strong performance with fewer images than anticipated. The quality of a portable supine CXR has been known to be highly variable because of differences in image exposure and scattered radiation, leading to poor NGT visibility [10, 38]. As the esophageal portions of NGTs are embedded within the mediastinum, the silhouette of surrounding structures such as the heart may further reduce NGT visibility with similar radiopacity. In addition, portable supine CXR may contain various overlaps or interconnections between NGTs and other tubes commonly used in critically ill patients, such as ETT or CVC. All these factors contribute to the substantial difficulty in accurate NGT detection, not only in clinical practice [10, 38] but also in the annotation. Hence, to facilitate annotation and model development, CLAHE [39] was employed to enhance the image contrast details and avoid noise amplification caused by histogram equalization. As shown in Table 4, the kappa value for NGT malposition was 0.821, indicating substantial inter-annotator agreement [37].

### Dataset Construction

As NGTs may be easily overlooked on portable supine CXR [10, 38] and not mentioned in clinical radiology reports, using NGT as a keyword to search for candidate images may inadvertently select only those with an easily visible NGT into analysis, resulting in selection bias. In our candidate negative group, the keywords ETT, NGT, and CVC were all used to establish the candidate list. As NGT was frequently accompanied by the placement of ETT or CVC on portable supine CXR among critically ill patients, using a combination of these keywords for random sampling may have increased our chances of including images with various radiological appearances of NGTs on CXR, regardless of whether or not they were mentioned in the report. As shown in Fig. 1, the number of images with NGT malposition was more in the annotated than the candidate positive groups. This gap may be explained by the lack of keywords in the clinical reports. Hence, a multi-keyword-based search strategy may have a higher chance of obtaining datasets with less selection bias.

We randomly selected images from different time periods (National Taiwan University Hospital-20) and locations (National Taiwan University Hospital-YB). Additionally, for these two testing datasets, we included only images taken in the ED. Table 1 highlights significant differences among these datasets, indicating their suitability for evaluating the external generalizability of the CAD system.

### Performance of Segmentation Model

Our CAD system could segment the NGT, NGT tip, lungs, and diaphragm. By visualizing the complete path of the NGT, including the tip, it would be easier for clinicians to verify the detection results. Tracing NGTs is not easy, as the NGT may loop on itself or take other aberrant courses. NGTs may also be confused with other similar linear structures, such as CVCs. Therefore, we used several approaches to improve the segmentation of the NGT. First, CLAHE intensified linear structures in images for a clear segmentation subject. Subsequently, the segmentation masks were morphologically dilated during training for a smoother loss, since thin-line structures such as an NGT yield an oversensitive loss. Lastly, tip attention was applied using spatial-weighted Dice loss, as the portion of NGT closer to the tip was more challenging to segment. The segmentation results of the Dice coefficient and NGT tip-tip distance were critical for the subsequent classification model to detect NGT malposition.

Besides delineating the NGT course, the CAD system could also segment out the lung and diaphragm. Based on the relative positions between the NGT tip, diaphragm, and lung, the classification model could determine whether the NGTs were malpositioned. It may be a concern that the low Dice coefficients for the diaphragm were not adequate for the classification tasks. However, suboptimal Dice coefficients for the diaphragm should not be surprising, as the portion where the diaphragm adjoins the lower heart border may be difficult to discern radiologically, rendering the ground truth difficult to be annotated and learned by the algorithm.

### Performance of Classification Model in the National Taiwan University Hospital-20 and National Taiwan University Hospital-Yunlin Branch Testing Datasets

The detection task was split into two stages to evaluate its performance. In the first stage, the classification model would detect the presence of NGT, demonstrating excellent performance with AUCs above 0.99 (Table 3). In the second stage, the model identified NGT malposition in the images where an NGT was present. In this study, NGT malposition was defined as bronchial or esophageal insertion, as it is crucial to ensure that the distal side holes of the NGT are positioned in the stomach to prevent aspiration. The AUCs of our CAD system for detecting NGT malposition were 0.964 and 0.991 in National Taiwan University Hospital-20 and National Taiwan University Hospital-Yunlin Branch, respectively, demonstrating the CAD algorithm’s excellent performance and consistent external generalizability. In a previous study, the Singh et al. [16] model could detect bronchial insertion with an AUC of 0.87. Bronchial insertion may cause more harm to patients than esophageal insertion and necessitate immediate attention and adjustment. Our analysis demonstrated that the performance of the CAD system was similarly high in detecting bronchial or esophageal insertion, with AUCs above 0.96.

Our study had a lower number of images with NGT malposition, with one (1.2%) and six (5.1%) in National Taiwan University Hospital-20 and National Taiwan University Hospital-Yunlin Branch, respectively. Since there are many other methods that healthcare providers could use to check the NGT position before obtaining a CXR, the number of images with malposition is expected to be small. Previous studies [40, 41] have reported that daily CXRs reveal about 0.3–0.4% of NGT malposition in ICUs. Therefore, the low prevalence of NGT malposition in our testing datasets may just reflect real-world settings.

### Performance in the CLiP Dataset

To the best of our knowledge, the CLiP dataset was the only public dataset with misplaced tubes annotated for external testing of our model. In the CLiP dataset, the classification model could detect the presence of nasoenteric tubes with similar performance as in NTUH-20 and NTUH-YB datasets for NGT. However, the model’s performance in detecting malpositioned nasoenteric tubes slightly decreased. The different tube types may cause the differences in performance. The CLiP dataset contained CXRs with nasoduodenal and nasojejunal tubes, which were longer than NGT. The proximal parts of the NGT and nasoduodenal and nasojejunal tubes may appear similar on CXRs but differ substantially distal to the gastroesophageal sphincter. The tube tip was important for the model to determine malposition. The different tube types may thus lead to a less favorable performance in detecting malpositioned nasoenteric tubes. Finally, in the CLiP dataset, each patient contributed eight CXRs to the dataset on average. These correlated images may lead to over- or underestimated classification performance, which could not be accounted for because the dataset did not offer the source information of these images. Given the limitations of the CLiP dataset, the external generalizability of the classification model may be considered good to excellent [42, 43]. Also, the classification model reached negative predictive value above 0.990 in all three testing datasets, which may be useful in confirming the tube position.

### Future Applications

Yi et al. [44] suggested that a clinically effective tube assessment model should be capable of performing five tasks: (1) detecting the presence of the tube, (2) localizing its tip, (3) tracing the tube’s course, (4) identifying the tube, and (5) determining whether the tube is correctly positioned. Previous studies [11–13, 16] have addressed some of these tasks but have not provided all the necessary information. To advance this goal, we employed a sequential inference strategy that integrates deep learning–based segmentation and classification models, providing clinicians with the most comprehensive results. The CAD system can be utilized to (1) prioritize CXRs, highlighting those requiring immediate review by the radiologist, or (2) send notifications to treating clinicians. When clinicians review the classification results, the segmentation masks of the NGT, lung, and diaphragm can be displayed to aid in verifying the findings.

### Study Limitations

First, the datasets were inherently imbalanced, as clinical protocols had already been established for checking NGT positioning, which may have reduced the number of malpositioned NGTs detected on CXRs. Second, the testing datasets only included images obtained in EDs. Most portable CXRs obtained in ICUs are used to follow pulmonary disease [40, 41], while those filmed in EDs are more likely to be used to check the position of a newly placed NGT. Random sampling of portable CXRs obtained in EDs may increase the probability of obtaining images with NGT and NGT malposition.

## Conclusions

The developed deep learning–based CAD system effectively localizes NGTs and identifies any malposition on portable supine CXRs taken in the ED and ICU. The consistent performance observed across different time periods and locations indicates that the system has strong potential for external generalizability.

## Supplementary Information

Below is the link to the electronic supplementary material.

Supplementary file1 (DOCX 16 KB)

## Data Availability

The data that support the findings of this study are available from the corresponding author upon reasonable request.

## Declarations

### Disclaimer

National Taiwan University Hospital and National Science and Technology Council had no involvement in designing the study; collecting, analyzing, or interpreting the data; writing the manuscript; or deciding whether to submit the manuscript for publication.
