# Supplementary material for: Deep Learning–Based Localization and Detection of Malpositioned Nasogastric Tubes on Portable Supine Chest X-Rays in Intensive Care and Emergency Medicine: A Multi-center Retrospective Study
Source: J Imaging Inform Med. 2024 Jul 9;38(1):335–45. doi: 10.1007/s10278-024-01181-z (PMC11811315; doi:10.1007/s10278-024-01181-z)
Supplement: Supplementary file 1 — Supplementary file1 (DOCX 16 KB) [file 10278_2024_1181_MOESM1_ESM.docx]

Supplemental Table 1. Keywords used in searching for candidate positive images for nasogastric tube malposition

| Search strategy | Keywords |
| --- | --- |
| Nasogastric tube malposition | “tip in esophagus,” “in the right bronchus," "in the left bronchus," "tip in right bronchus," "NG looping," "tip in upper esophagus," "tip in middle esophagus," "tip in lower esophagus," "looped nasogastric tube," "looping nasogastric tube," "NG tube looping," "looping NG," "looped NG," "nasogastric tube looping," "nasogastric tube looped," "NG proximal migration," "looping of," "malplacement," "misplacement," "tip position." |
